# DNA chip-assisted diagnosis of a previously unknown etiology of intermediate uveitis – *Toxoplasma gondii*

**DOI:** 10.4103/0301-4738.71714

**Published:** 2010

**Authors:** Soumyava Basu, Savitri Sharma, Sarita Kar, Taraprasad Das

**Affiliations:** Retina-Vitreous Service India; 1 Ocular Microbiology Service, LV Prasad Eye Institute, Bhubaneswar, Orissa, India

**Keywords:** Intermediate uveitis, *Toxoplasma gondii*, polymerase chain reaction

## Abstract

We report the use of DNA chip technology in the identification of *Toxoplasma gondii* as the etiological agent in two patients with recurrent intermediate uveitis (IU). Both patients had recurrent episodes of vitritis (with no focal retinochoroidal lesion) over varying time intervals and were diagnosed to have IU. The tuberculin test was negative in both. Blood counts, erythrocyte sedimentation rate, and serum angiotensin convertase enzyme levels were normal. In both cases, the vitreous fluid tested positive for the *T. gondii* DNA sequence by using a uveitis DNA chip (XCyton Pvt. Ltd., Bangalore, India). It contained complimentary sequences to “signature genes” of *T. gondii, Mycobacterium tuberculosis, M. chelonae*, and *M. fortuitum*. The enzyme-linked immunosorbent assay (ELISA) detected elevated serum antitoxoplasma IgG levels in both. They responded to the antitoxoplasma therapy with oral co-trimoxazole (and additional intravitreal clindamycin in patient 1), with no recurrence during follow-ups of 6 and 8 months, respectively.

Focal necrotizing retinitis, with an overlying vitreous inflammatory haze is considered characteristic of ocular toxoplasmosis.[1] However, atypical manifestations like neuroretinitis, punctate outer retinal toxoplasmosis, retinal vasculitis, and acute retinal necrosis have been reported.[2] We report two cases where *Toxoplasma gondii* was identified as the etiological agent for recurrent intermediate uveitis (IU), using DNA chip technology.

## Case Reports

### Case 1

A 56-year-old man presented to us with recurrent episodes of reduced vision and floaters in the right eye for the past 5 months. He had been diagnosed as IU elsewhere and was treated with oral steroids. The inflammation would recur, each time oral steroids were tapered to 15–20 mg/day. Complete blood counts, tuberculin reaction, serum angiotensin convertase enzyme (ACE) levels, and chest X-ray were normal. On examination, the right eye had a best-corrected visual acuity of counting fingers at 2 m, significant anterior chamber reaction, and vitreous inflammatory haze, through which only the disk could be hazily seen. Left eye findings were normal.

**Figure 1:**
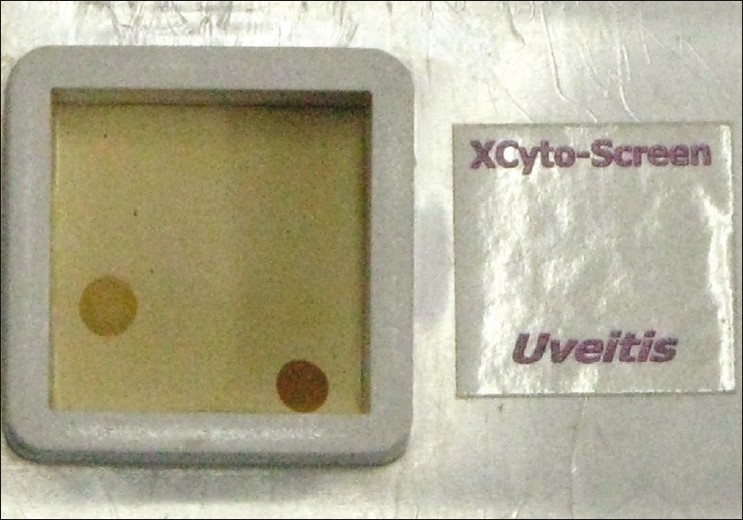
The DNA chip, showing a positive reaction at the position of *T. gondii* (B1 gene sequence) and the positive control (β-globin gene)

Tuberculosis and sarcoidosis were considered unlikely etiologies, as tuberculin test, serum ACE levels, and chest X-ray were normal. The possibility of a masquerade syndrome was not investigated further, considering the complete response to steroids on previous occasions and rarity of the condition. Since the disease followed a pattern of multiple recurrences on tapering of steroids, we suspected an underlying infectious etiology. A vitreous biopsy was done for DNA amplification using the multiplex polymerase chain reaction (PCR) technique. To identify the amplified DNA, we used a DNA chip (XCyton Diagnostics Pvt. Ltd., Bangalore, India; Fig. 1), containing complimentary sequences to “signature genes” of *Toxoplasma gondii, Mycobacterium tuberculosis, M. chelonae*, and *M. fortuitum* [Table 1]. We got a positive reaction for the B1 gene sequence of *T. gondii* [Fig. 1]. Subsequently, the enzyme-linked immunosorbent assay (ELISA) detected very high antitoxoplasma IgG levels. Oral steroids were stopped. We advised oral co-trimoxazole (960 mg twice daily) and a single dose of intravitreal clindamycin (1 mg/0.1 ml). This led to a sharp reduction in vitreous inflammation and an improvement in the visual acuity to 20/50 after 2 weeks. No focal lesions were seen in the fundus. There was no recurrence over the next 6 months of follow-up.

**Table 1:** Schematic representation of the DNA chip for uveitis, showing the position of gene probes for different organisms

*M. tuberculosis* (MPB 64 gene)	blank	*M. chelonae* (16-23S rRNA)
Blank	*M. fortuitum* (16-23S rRNA)	blank
*T. gondii* (B1 gene)	blank	blank
blank	blank	Positive control (β globin gene)

### Case 2

A 28-year-old lady presented with reduced vision in both eyes for 1 month. On examination, her best-corrected visual acuity was 20/50 in the right eye and 20/30 in the left eye. The anterior segment showed grade 1 cells in both eyes. A vitreous inflammatory haze with cotton ball exudates in inferior fundus was seen in both eyes. No focal lesions were seen. Tests for tuberculin reactivity and sarcoidosis were normal. She did not give any history of contact with pets (to rule out bartonellosis). She was not investigated further and a diagnosis of idiopathic IU in both eyes was made. She was treated with oral and periocular corticosteroids, following which the vitreous haze reduced and vision improved in both eyes. Oral corticosteroids were tapered over 3 months. However, 1 month after stopping corticosteroid therapy, she had a recurrence of previous symptoms and similar findings were noted. She was again treated with periocular and oral corticosteroids, but had two more recurrences over the next 8 months. After the third recurrence, serum antitoxoplasma IgG levels were tested using ELISA, and found to be mildly raised. Subsequently, a vitreous biopsy from the right eye, followed by multiplex PCR and DNA chip identification revealed positive reaction for *T. gondii*. She was treated with intravitreal clindamycin (single dose) and oral co-trimoxazole, but needed additional oral steroids, for vitreous inflammation to subside. Visual acuity improved to 20/30 and 20/20 in right and left eyes, respectively. No recurrence was seen over the next 8 months of follow-up.

## Discussion

This report highlights the use of DNA chip technology in the diagnosis of a previously unknown etiology for IU. The role of PCR in infectious uveitis has been documented before.[3] The application of multiplex PCR (simultaneous amplification of multiple gene fragments) in infectious uveitis has also been validated.[4] The DNA chip used in our study, had complimentary sequences to “signature genes” of four different organisms. The genes were selected on the basis of high sensitivity and specificity of uniplex PCR in the diagnosis of specific infectious uveitis, using primers for the same genes. The DNA chip was further validated using positive and negative controls from standard strains as well as patient samples (HN Madhavan, personal communication).

Intraocular inflammatory reactions due to *T. gondii*, in the absence of focal retinochoroiditis, have been reported earlier in serologically proven cases of acquired systemic toxoplasmosis.[5] Such inflammatory reactions are attributed to the host immune response to parasitic antigens, whereas focal lesions are caused by live intraretinal parasites. To our knowledge, this is the first PCR-proven report of an intraocular inflammatory reaction resembling IU, caused by *T. gondii* (PubMed search). The good response to the antitoxoplasma therapy and the lack of recurrence in both cases also favor *T. gondii* as the causative agent. The demonstration of local antibody production, using the Goldman–Witmer coefficient in these patients could have provided additional evidence.

To summarize, our report showed that multiplex PCR-based DNA chip technology can be used to investigate a previously unsuspected etiology in IU, particularly recurrent cases. It also shows that the vitreous inflammatory haze/IU can be the sole manifestation of ocular toxoplasmosis.
